# Assessment of BMP responses in an *in vitro* model of acute ethanol toxicity

**DOI:** 10.1016/j.mex.2022.101631

**Published:** 2022-02-06

**Authors:** Naila Habeeb, Sheyda Najafi, Jeanette C. Perron

**Affiliations:** Department of Pharmaceutical Sciences, College of Pharmacy and Health Sciences, St. John's University

**Keywords:** BMPs, Bone Morphogenetic Proteins, CGM, Complete Growth Medium, DRG, Dorsal Root Ganglion, EtOH, Ethanol, IF, Immunofluorescence, PAE, Prenatal Alcohol Exposure, PI3K, Phosphatidylinositol 3-kinase, PS, Penicilin/Streptomycin, PSG, Penicilin/Strepomycin/Glutamine, SSM, Serum starvation medium, Western blot, Immunofluorescent labeling, Smad, PI3K/Akt, C2C12 cells, DRG neurons

## Abstract

The assay presented here was designed to assess the immediate effects of ethanol (EtOH) exposure on intracellular signaling activated by BMPs (Bone Morphogenetic Proteins). Previous reports of the relationship between EtOH exposure and BMP-dependent signaling have primarily assessed the expression of individual BMPs, changes in BMP target genes or effects on the phosphorylation level of key downstream mediators after days or weeks of *in vivo* EtOH exposure. What happens to BMP-stimulated signaling immediately following exposure to EtOH remains largely unexplored. Here, the early events of BMP-evoked intracellular signaling were examined in an *in vitro* model of acute EtOH toxicity. The BMP/Ethanol Stimulation Assay involved first stimulating cultured cells with recombinant BMPs. BMP-evoked intracellular signaling was then allowed to develop for 30 minutes. Next, the cells were exposed to a range of EtOH concentrations for an additional 30 minutes. Finally, the cultures were processed for Western blot analysis or immunofluorescent labeling.

This short-term assay:

• Permits investigation of EtOH exposure during the initial signaling events downstream of BMP receptor activation

• Enables assessment of how the presence of BMPs might protect against cellular injury caused by toxic EtOH levels

## Graphical abstract

**Figure:**
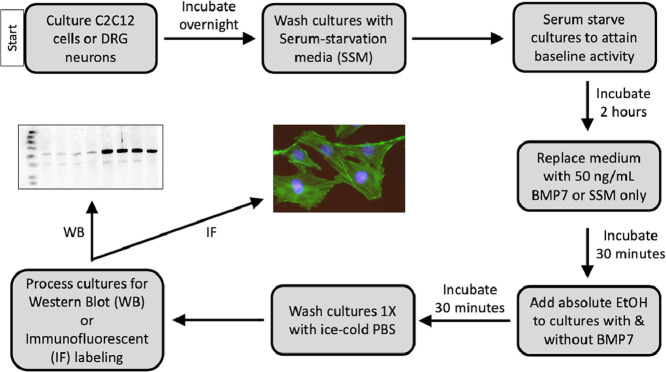

Specifications Table

**Table:** 

Subject Area;	Pharmacology, Toxicology and Pharmaceutical Science
More specific subject area;	Acute Ethanol Toxicity
Method name;	BMP/Ethanol Stimulation Assay
Name and reference of original method;	*Phosphorylation Assays:* Perron JC, Rodrigues AA, Surubholta N, Dodd J. Chemotropic signaling by BMP7 requires selective interaction at a key residue in ActRIIA. Biol Open. 2019 Jul 16;8(7):bio042283. doi: 10.1242/bio.042283
Resource availability;	Recombinant BMP7 (R&D Systems – catalog #5666-BP carrier free) Phospho-Smad1/5/8 (D5B10) Rabbit mAb (Cell Signaling Technology – catalog #13820) Phospho-Akt (Ser473) (D9E) Rabbit mAb (Cell Signaling Technology – catalog #4060)

The following protocol describes the BMP/EtOH stimulation assay (Fig. 1) performed with the multipotent BMP-responsive C2C12 myoblast cell line. The C2C12 cells were maintained in T75 flasks in Complete Growth Medium (CGM) containing Dulbecco's Modified Eagle's Medium (DMEM), 1X Penicillin/Streptomycin/Glutamine solution (PSG), and 10% Fetal bovine serum (FBS). The same steps were followed after culturing primary DRG neurons dissected from embryonic day 16 (E16) mouse embryos. DRGs were dissociated and cultured in 24-well plates on poly-D-lysine/laminin-coated glass coverslips at a density of 2.5 × 10^4^ cells/well in Neurobasal medium, 1X B-27 supplement, 35 mM glucose and PSG. All cultures were incubated at 37°C in 5% CO_2_.

**Fig. 1:**
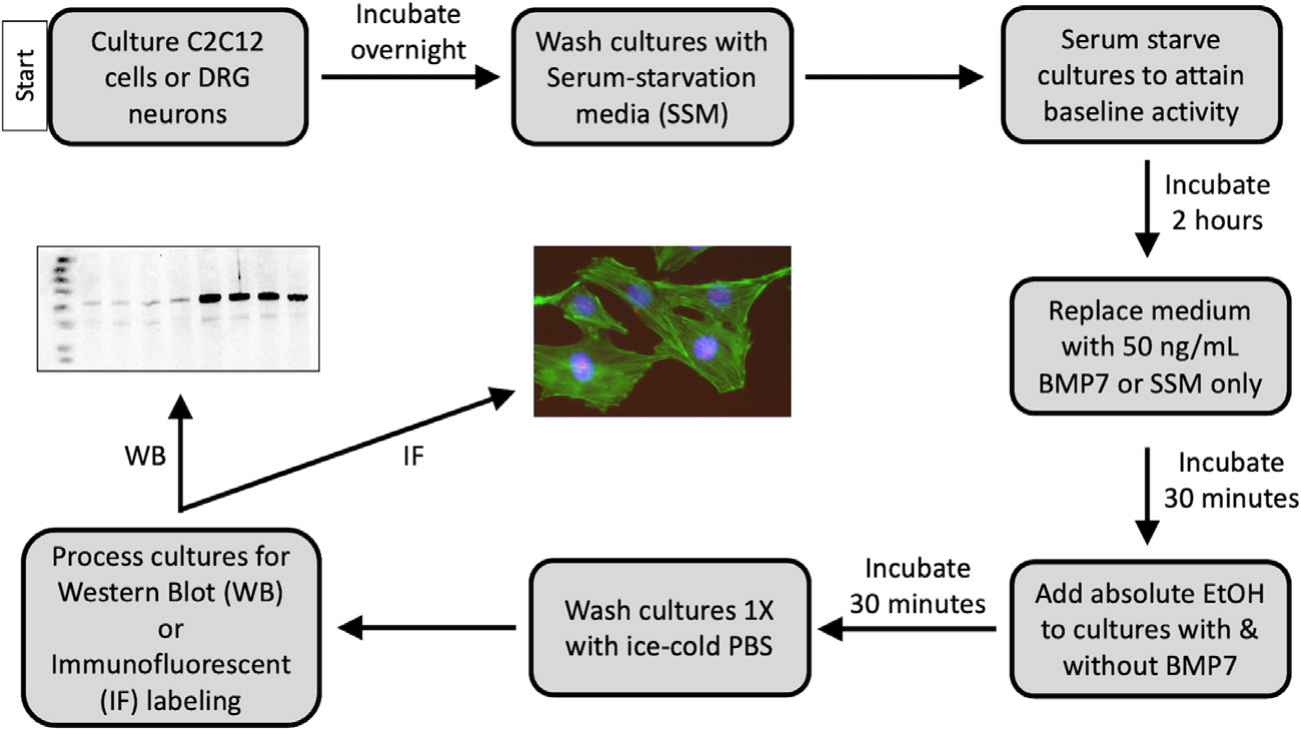
Scheme for BMP/EtOH Stimulation Assay.

## BMP/ethanol stimulation assay

1. Seed C2C12 cells in 35 mm tissue culture dishes for Western analysis at a density of 1 × 10^5^ cells/mL (3 mL/dish) and in 24-well plates for immunofluorescence (IF) labeling at a density of 1 × 10^5^ cells/mL (0.5 mL/well) on 12 mm glass coverslips coated with 0.1 mg/mL poly-D-Lysine in CGM and incubate for approximately 24 hours.
2. Cultures should be ∼90% confluent for preparing whole cell lysates and ∼70-80% confluent for IF labeling.
3. On the day of the assay, prepare Serum Starvation Medium (SSM) using low glucose DMEM supplemented with 1X Penicillin/Streptomycin solution (PS).
4. Aspirate CGM and wash the cultures once with 2 mL fresh SSM by slowly pipetting the medium down the side of the dish or well.
5. To ensure efficient washing of the cultures, move the dish or plate in a figure eight motion to evenly distribute the medium across the culture.
6. Aspirate the SSM from the washing step and add 2 mL fresh SSM to each culture dish or well.
7. Incubate the cultures in SSM for 2 hours to ensure adequate serum-starvation. Longer incubation times were determined not necessary for ensuring low, baseline levels of either Smad or Akt phosphorylation in either C2C12 or DRG cultures.
8. Recombinant BMP7 (R&D Systems) was reconstituted in 4 mM HCl and prepared as a stock solution at a concentration of 100 µg/mL.
  a Stock solutions of BMP7 were kept in 5 µL aliquots at -20°C.
  b BMP7 aliquots were thawed on ice just prior to use.
9. Aspirate the SSM and replace with freshly prepared treatment media by adding 2 mL to 35 mm dishes and 0.5 mL to 24-well plates.
  a For the negative control, add SSM only.
  b For BMP7 treatment, add 50 ng/mL BMP7 in SSM.
  c Carefully pipet the treatment media down the sides of the culture vessel to minimize dislodging the cells.
  d In order to ensure media is thoroughly dispersed, move the vessel gently in a figure eight motion.
10. Incubate the cultures for 30 minutes at 37°C in 5% CO_2_.
11. The necessary volumes of absolute EtOH were then added directly to the cultures containing the SSM or BMP7/SSM solutions to achieve final concentrations of 2.5%, 5%, 7.5% and 10% EtOH.
  a Work quickly and keep the culture vessel covered as much as possible to minimize evaporation of EtOH.
  b Pipet EtOH down the sides of the vessel to minimize dislodging the cells.
  c Move the vessel gently in a figure eight motion to ensure media is thoroughly dispersed.
12. Incubate for 30 minutes at 37°C in 5% CO_2_.
13. Following the final incubation, place the cultures on ice. Carefully replace the medium with ice-cold PBS and proceed to preparation of whole cell lysates for Western blot analysis or paraformaldehyde-fixation for IF labeling.

## Method validation

**Fig. 2:**
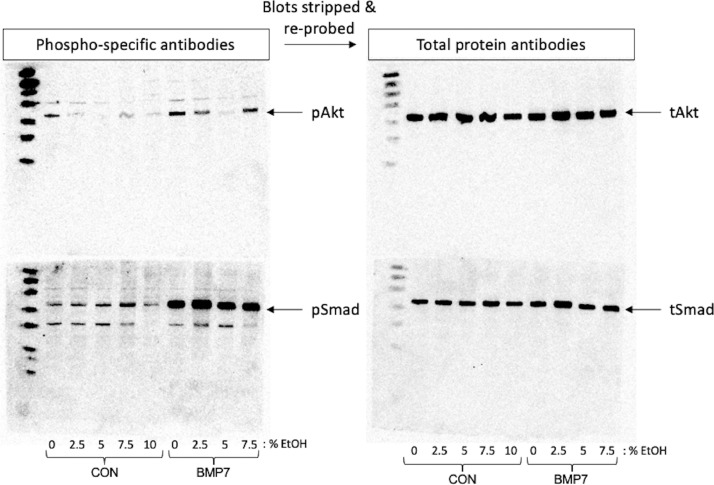
Representative Western blot data. Whole cell lysates of C2C12 cultures treated with or without 50 ng/mL BMP7 followed by exposure to the indicated percentages of EtOH were separated on SDS-PAGE and transferred to nitrocellulose membranes as described [3]. The membranes were probed with phospho-specific antibodies for Akt (top, left) and Smad1/5/8 (bottom, left). Next, the membranes were stripped in mild stripping solution (200 mM glycine/0.1% SDS/1% Tween 20 pH 2.2) and re-probed with antibodies against total Akt (top, right) and total Smad (bottom, right). The blots were developed using GeneTex HRP substrate solutions and analyzed by capturing the chemiluminescent signal using the Omega Lum™ G Imaging System.

**Fig. 3:**
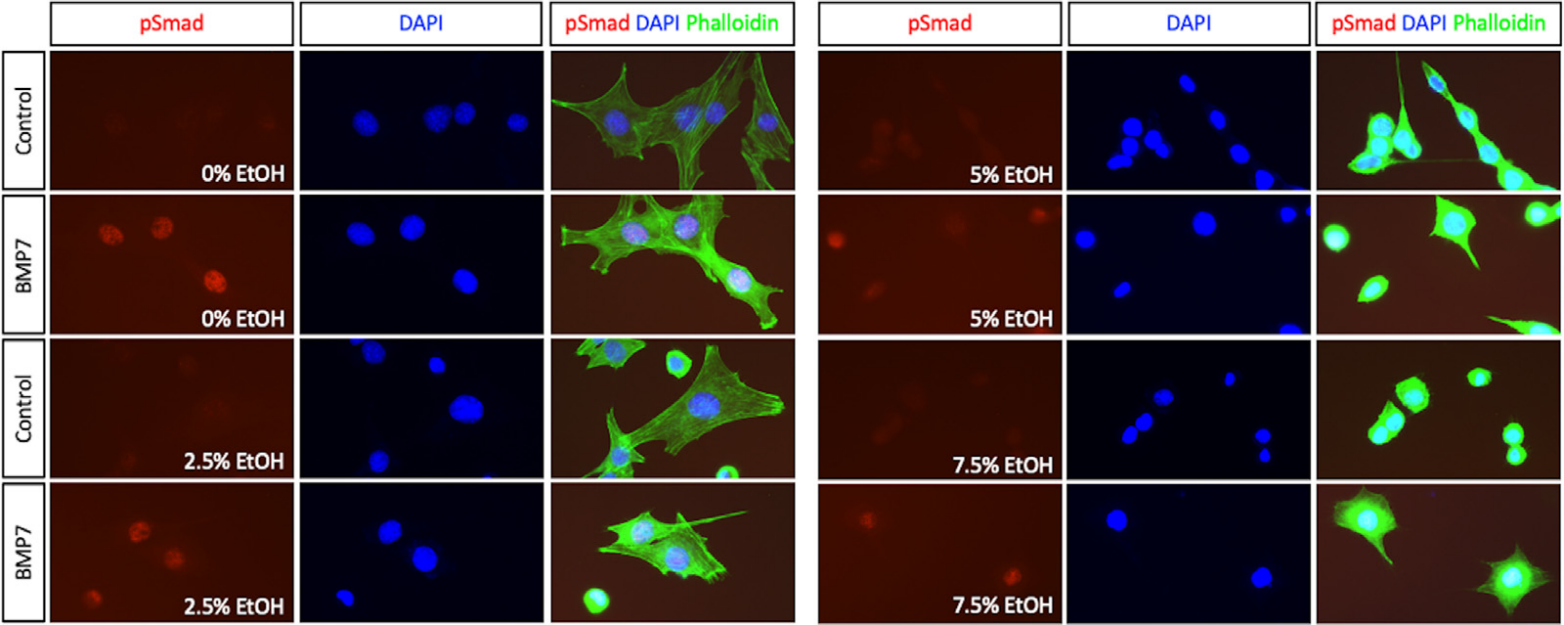
Representative images of Immunofluorescent labeling. C2C12 cultures treated with or without 50 ng/mL BMP7 followed by exposure to the indicated percentages of EtOH were fixed in 4% paraformaldehyde/PBS and labeled with anti-phospho-Smad antibodies as described [5]. Alexa Fluor 488 Phalloidin (Invitrogen) was included in the secondary antibody step to label actin filaments. DAPI was used to stain the nucleus and was present in the mounting medium (Vectashield Antifade Mounting Medium with DAPI). Images were captured using a Zeiss Axioplan 200M upright fluorescent microscope, AxioCam HRm digital camera and AxioVision 4.8.2.0 software.

If a pregnant mother consumes excessive quantities of alcohol, BMP-mediated intracellular signaling would be actively influencing cellular differentiation, tissue patterning and many other aspects of embryonic development [1,2]. Thus, in the BMP/EtOH Stimulation Assay, EtOH exposure occurs after the cells are stimulated with BMP7. The BMP signaling pathway has divergent signaling arms: one responsible for cytoskeletal rearrangement through the PI3K/Akt and LIMK signaling pathways and others for gene transcription through primarily Smad- and p38-dependent pathways [3,4]). Using the protocol described here, phosphorylation levels of Smad and Akt (pSmad and pAkt, respectively) were monitored by both Western blot analysis (Fig. 2) and Immunofluorescent (IF) labeling (Fig. 3). A list of the primary and secondary antibodies used in the experiments are provided in Table 1.

**Table 1:** Antibodies and respective dilutions for Western blots and IF labeling.

Western Blotting	
Primary Antibody	Dilution
Phospho-Smad1/5/8 (D5B10) Rabbit mAb (Cell Signaling Technology – catalog #13820)	1:4000
Phospho-Akt (Ser473) (D9E) Rabbit mAb (Cell Signaling Technology – catalog #4060)	1:8000
Smad1 (D59D7) Rabbit mAb (Cell Signaling Technology – catalog #6944)	1:4000
Akt (pan) (C67E7) Rabbit mAb (Cell Signaling Technology – catalog #4691)	1:50000
**Secondary Antibody**	
HRP-conjugated mouse anti-rabbit IgG (Santa Cruz Biotechnology – catalog #sc-2004)	1:10000
**Immunolabeling**	
**Primary Antibody**	
Phospho-Smad1/5/8 (D5B10) Rabbit mAb (Cell Signaling Technology – catalog #13820)	1:400
Phospho-Akt (Ser473) (D9E) Rabbit mAb (Cell Signaling Technology – catalog #4060)	1:400
**Secondary Antibody**	
Cy3 goat anti-rabbit IgG (Jackson ImmunoResearch Laboratories – catalog #111-165-144)	1:500
**Cell Staining**	
Alexa Fluor 488 Phalloidin (Invitrogen – catalog #A12379)	1:500
Vectashield Antifade Mounting Medium with DAPI (Vector Laboratories – catalog #H1200)	

The Western blot analyses were carried out by simultaneously probing for both Smad and PI3K/Akt pathway activation in whole cell lysates derived from BMP7/EtOH-treated C2C12 cell cultures (Fig. 2). Two gels were loaded with identical samples, separated by electrophoresis and transferred to nitrocellulose membranes. The membranes were first probed with anti-pSmad and anti-pAkt primary antibodies followed by incubation with HRP-conjugated goat anti-rabbit secondary antibodies. After visualization of the phospho-specific signals by enhanced chemiluminescence, the membranes were stripped and re-probed with antibodies that recognize total Smad and total Akt protein in the lysates. This approach allows simultaneous monitoring of distinct arms of BMP-stimulated signaling pathways in the same whole cell lysate sample for each replicate. Fig. 2 demonstrates that while pSmad levels are not affected by treatment with increasing concentrations of EtOH, the levels of pAkt are significantly impacted by exposure to EtOH. Including the blots probed for total Smad or total Akt protein ensures that the changes observed for the phosphorylated proteins are not the result of uneven protein loading or changes to the cellular proteins by the various treatments.

Upon activation of the Smad pathway, Smad proteins are not only phosphorylated by BMP receptors, but these proteins are also required to translocate to the nucleus in order to transcribe BMP target genes. IF labeling of BMP7/EtOH-treated C2C12 cultures with anti-pSmad antibodies validates the Western blot data, which showed no change in pSmad after exposure to EtOH (Fig. 3). Moreover, nuclear translocation of pSmad was assessed by staining the cultures with DAPI. Furthermore, co-staining actin filaments with Phalloidin allowed for assessment of the subcellular architecture of BMP7-stimulated cells exposed to high concentrations of EtOH. The combination of Western blot and IF data permits analysis of the divergent BMP signaling pathways following the BMP/EtOH Stimulation Assay.

## Declaration of Competing Interest

The authors declare that they have no known competing financial interests or personal relationships that could have appeared to influence the work reported in this paper.

## References

[1] May P.A., Blankenship J., Marais A.S., Gossage J.P., Kalberg W.0., Joubert B., Cloete M., Barnard R., De Vries M., Hasken J., Robinson L.K., Adnams C.M., Buckley D., Manning M., Parry C.D., Hoyme H.E., Tabachnick B., Seedat S. (2013 Dec 1). Maternal alcohol consumption producing fetal alcohol spectrum disorders (FASD): quantity, frequency, and timing of drinking. Drug Alcohol Depend..

[2] Wang R.N., Green J., Wang Z., Deng Y., Qiao M., Peabody M., Zhang Q., Ye J., Yan Z., Denduluri S., Idowu O., Li M., Shen C., Hu A., Haydon R.C., Kang R., Mok J., Lee M.J., Luu H.L., Shi L.L. (2014 Sep). Bone Morphogenetic Protein (BMP) signaling in development and human diseases. Genes Dis.

[3] Perron J.C., Rodrigues A.A., Surubholta N., Dodd J. (2019 Jul 16). Chemotropic signaling by BMP7 requires selective interaction at a key residue in ActRIIA. Biol. Open.

[4] Derynck R., Budi E.H. (2019 Feb 26). Specificity, versatility, and control of TGF-β family signaling. Sci. Signal.

[5] Perron J.C., Dodd J. (2011 Nov 15). Inductive specification and axonal orientation of spinal neurons mediated by divergent bone morphogenetic protein signaling pathways. Neural. Dev..

[6] Ornoy A., Ergaz Z. (2010 Feb). Alcohol abuse in pregnant women: effects on the fetus and newborn, mode of action and maternal treatment. Int. J. Environ. Res. Public Health.

[7] Sarmah S., Muralidharan P., Marrs J.A. (2016 Aug 24). Embryonic Ethanol Exposure Dysregulates BMP and Notch Signaling, Leading to Persistent Atrio-Ventricular Valve Defects in Zebrafish. PLoS One.

[8] Zhang P., Wang G., Lin Z., Wu Y., Zhang J., Liu M., Lee K.K.H., Chuai M., Yang X. (2017 Nov 5). Alcohol exposure induces chick craniofacial bone defects by negatively affecting cranial neural crest development. Toxicol. Lett..

[9] Nickel J., Mueller T.D. (2019 Dec 5). Specification of BMP Signaling. Cells.

[10] Dolganiuc A., Szabo G. (2009 Mar 14). In vitro and in vivo models of acute alcohol exposure. World J. Gastroenterol..

[11] Habeeb N., Najafi S., Perron J.C. (2021 Dec 15). Non-Smad, BMP-dependent signaling protects against the effects of acute ethanol toxicity. Toxicol. Lett..

